# Spontaneous neural activity in the three principal networks underlying delay discounting: a resting-state fMRI study

**DOI:** 10.3389/fpsyt.2024.1320830

**Published:** 2024-02-02

**Authors:** Songyue Ji, Fan Yang, Xueting Li

**Affiliations:** Department of Psychology, Renmin University of China, Beijing, China

**Keywords:** delay discounting, ReHo, functional connectivity, the tripartite network model, resting-state fMRI

## Abstract

Delay discounting, the decline in the subjective value of future rewards over time, has traditionally been understood through a tripartite neural network model, comprising the valuation, cognitive control, and prospection networks. To investigate the applicability of this model in a resting-state context, we employed a monetary choice questionnaire to quantify delay discounting and utilized resting-state functional magnetic resonance imaging (rs-fMRI) to explore the role of spontaneous brain activity, specifically regional homogeneity (ReHo), in influencing individual differences in delay discounting across a large cohort (*N* = 257). Preliminary analyses revealed a significant negative correlation between delay discounting tendencies and the ReHo in both the left insula and the right hippocampus, respectively. Subsequent resting-state functional connectivity (RSFC) analyses, using these regions as seed ROIs, disclosed that all implicated brain regions conform to the three principal networks traditionally associated with delay discounting. Our findings offer novel insights into the role of spontaneous neural activity in shaping individual variations in delay discounting at both regional and network levels, providing the first empirical evidence supporting the applicability of the tripartite network model in a resting-state context.

## 1 Introduction

Consider two everyday dilemmas: You’re aware of the long-term health benefits of physical exercise, yet the immediate allure of a riveting television show or an engaging video game keeps you anchored to the couch. Or, you’re a student facing an impending exam that could shape your academic future, but the temptation to socialize or dive into another round of gaming is strong. Given the choice, will you favor the immediate satisfaction or defer gratification for future gains? Often, when faced with a smaller, immediate reward (SIR), such as a riveting television show or another round of gaming, and a larger, delayed reward (LDR), such as health benefits from exercise or enhanced academic future, we tend to opt for the former. This tendency is termed delay discounting, a psychological construct defined by the diminishing subjective value of a reward as the waiting period increases (1). Delay discounting is found to be associated with a plethora of unhealthy behaviors, including drug addiction (2), obesity (3), internet addiction (4), gambling (5), and alcohol use disorder (6).

The systematic devaluation of future rewards is commonly understood to arise from the complex interplay among a tripartite neural network model: valuation, cognitive control, and prospection, as delineated in existing neuroscientific paradigms (7). The valuation network, composed of the ventral medial prefrontal cortex (vmPFC), medial orbitofrontal cortex (mOFC), ventral striatum (VS), and posterior cingulate gyrus (PCC), is considered crucial for valuation, with the insula playing a potential role (8, 9). The cognitive control network, encompassing the lateral prefrontal cortex (LPFC) and anterior cingulate gyrus (ACC), facilitates cognitive control, conflict monitoring, and strategy adaptation. The prospection network, primarily involving the medial temporal lobe areas like the hippocampus, facilitates future-oriented thinking. These networks are widely employed to explain delay discounting at the neural level (9–12). Nonetheless, the extent to which spontaneous neural activity relevant to delay discounting aligns with the theoretical underpinnings of the tripartite neural network model has yet to be elucidated. We posit that these principal neural networks, which are instrumental in governing delay discounting behavior, persist in their interactions during the resting state and may account for observed individual variances in delay discounting propensities.

Our research focuses on the regional homogeneity (ReHo) of spontaneous neural activity in a resting state. ReHo quantifies the temporal synchronicity between a specific voxel (a unit of three-dimensional space) and its neighboring voxels, serving as a metric for local functional connectivity. To test our hypothesis, we initially aimed to pinpoint brain regions implicated in delay discounting by examining the correlation between ReHo and delay discounting across each voxel in a substantial sample of participants (*N* = 257). Following the identification of salient brain regions, we employed these areas as seed regions to calculate whole-brain functional connectivity. This approach was designed to explore the interplay among neural networks at rest and elucidate their roles in shaping individual tendencies toward delay discounting.

## 2 Materials and methods

### 2.1 Participants

Our study enrolled 310 university students, ranging in age from 18 to 23 years (mean age = 20.36 years, *SD* = 0.85). Of these, 186 were females. Eight participants did not disclose their age. All participants had no reported history of neurological or psychiatric disorders. The study received approval from the Institutional Review Board of Beijing Normal University, and all participants provided informed consent before commencing the experiment.

### 2.2 Behavioral measures

#### 2.2.1 Delay discounting - money choice questionnaire

We employed the Money Choice Questionnaire to evaluate the level of delay discounting among participants. This questionnaire, composed of 27 items, prompts participants to choose between smaller immediate rewards and larger delayed rewards. An example item is, “Would you prefer to receive ¥31 immediately following the experiment, or ¥85 after 7 days?” The questionnaire did not impose a time limit.

To encourage participants to make choices reflective of their genuine preferences, each participant was informed they would not only receive a reward after the experiment, but also a randomly determined reward corresponding to one of their 27 choices. In our study, the Money Choice Questionnaire was utilized to classify delayed rewards into three categories: small (S), medium (M), and large (L). Each category is associated with 10 distinct choice patterns, each linked to a specific ‘*k*’ value. Participants were assigned a ‘*k*’ value that corresponded to the highest proportion of their choices aligning with that value. Essentially, for each participant, we calculated the proportion of their choices consistent with each of the 10 ‘*k*’ values defined by the questionnaire. The ‘*k*’ value that demonstrated the highest consistency with the participant’s choices was then assigned to them. To ensure the precision of our data, only participants who exhibited a consistency rate of 85% or higher in their responses were included in our analysis.

Although the participants’ delay discounting rates in our study were determined by aligning their choices with the 10 specific ‘*k*’ values, the significance of the ‘*k*’ value itself warrants a detailed explanation. The hyperbolic discounting model, expressed as V = A/(1 + k*D), is a foundational mathematical model in delay discounting research (13). In this formula, A represents the delayed reward, D the delay duration, and V the present value of reward A at delay D. This model delineates the reduction in subjective value of a future reward (V) as the delay (D) increases in relation to the actual reward value (A), where ‘*k*’ is an indicator of an individual’s delay discounting level. A higher ‘*k*’ value, for a given A and D, results in a lower present value (V), indicating greater levels of delay discounting and impulsivity. However, it is crucial to recognize that in the hyperbolic discounting model, the distribution of ‘*k*’ values is typically skewed (14–16). The subjective value V does not surpass the actual value A after a brief delay and becomes negative after an extended delay (14). Therefore, in our analysis, we employed the median as a measure of central tendency and applied a natural logarithm transformation to the ‘*k*’ values, ensuring a normal distribution of the calculated Ln(*k*) (17).

#### 2.2.2 Intelligence measurement - Raven’s advanced progressive matrices

Previous meta-analytic research has suggested a negative correlation between intelligence and delay discounting—higher intelligence associates with lower delay discounting (18). Moreover, fMRI studies have reported that greater self-control, reflected as lower delay discounting, correlates with higher intelligence (19). General intelligence may influence the preference for immediate rewards, thus reducing impulsivity in delay discounting tasks (20). To consider intelligence’s impact on delay discounting, we utilized Raven’s Advanced Progressive Matrices to measure participants’ intelligence. This test, comprising 36 items, requires participants to select the missing figure to complete a 3 × 3 matrix. We used the number of correct responses provided within a 30-minute window as the intelligence score.

### 2.3 Image acquisition

We collected imaging data using a Siemens MAGNETOM Trio 3T scanner equipped with a 12-channel phased-array head coil at the Brain Imaging Research Center, Beijing Normal University, Beijing, China. The resting-state scan included 240 consecutive echo-planar imaging (EPI) volumes (TR = 2000 ms; TE = 30 ms; flip angle = 90°; number of slices = 33; matrix = 64 × 64; FOV = 200 × 200 mm²; acquisition voxel size = 3.125 × 3.125 × 3.6 mm³). Additionally, we acquired high-resolution T1-weighted images using a magnetization-prepared gradient echo sequence (MPRAGE: TR/TE/TI = 2530/3.39/1100 ms; flip angle = 7°; matrix = 256×256; number of slices = 128; voxel size = 1 × 1 × 1.33 mm³) to facilitate spatial registration. During the scanning session, we instructed participants to close their eyes, maintain stillness, stay awake, and refrain from engaging in purposeful thinking.

### 2.4 Image data preprocessing

Resting-state fMRI data underwent preprocessing using the FMRI Expert Analysis Tool (FEAT Version 5.98), part of FMRIB’s Software Library (FSL, http://www.fmrib.ox.ac.uk/fsl). The preprocessing involved several steps: head motion correction (each volume was aligned to the central volume of the image series using MCFLIRT), spatial smoothing (applying a Gaussian kernel of 6-mm FWHM), intensity normalization, and linear trend removal. To mitigate the impact of physiological noise, including artifacts related to head motion, cardiac and respiratory cycles, our study employed a regression of 18 nuisance signals. These signals were derived from cerebrospinal fluid, white matter, global brain average, and motion correction parameters, as suggested in previous studies (21, 22). Specifically, our nuisance regressors encompassed the average cerebrospinal fluid signal, average white matter signal, global signal from the entire brain, six parameters from rigid-body head motion correction, and their derivatives (23). This approach in data preprocessing was meticulously chosen to address both linear and non-linear effects of head motion, thus substantially bolstering the reliability and validity of our study’s outcomes.

The registration of each participant’s resting-state fMRI to their anatomical images was accomplished using FMRIB’s Linear Image Registration Tool (FLIRT) to generate a 6 degree-of-freedom affine transformation matrix. The registration of each participant’s anatomical images to the Montreal Neurological Institute (MNI) space was accomplished by employing FLIRT to compute a 12 degree-of-freedom linear affine matrix (24, 25). In addition, considering the sensitivity of low-frequency fluctuations to spontaneous brain activity in gray matter regions (26), we defined a gray mask with a probability threshold of 0.5 in SPM8. The resulting gray mask incorporated a total of 128,190 voxels.

### 2.5 Statistical analysis

In our initial cohort, exclusions were made as follows: 37 participants due to incomplete scan data, 5 participants for demonstrating less than 85% consistency in the delay discounting task (17), 6 participants for lacking intelligence test data, 1 participant for missing age information, 1 participant for an intelligence score exceeding ±3 standard deviations, and 3 participants for head motion exceeding ±3 standard deviations. Following these exclusions, the final dataset consisted of 257 participants (157 females). Their ages ranged from 18 to 23 years (mean = 20.35, *SD* = 0.87), intelligence scores spanned from 15 to 34 (mean = 25.74, *SD* = 3.99). In our study, we employed Framewise Displacement (FD), measured in millimeters (mm), as the parameter for assessing head motion. Following the exclusion of participants with head motion exceeding ±3 standard deviations, as previously described, the head motion parameters for those included in the final dataset were as follows: The FD values ranged from 0.036 to 0.213 mm, with an average of 0.102 mm and a standard deviation of 0.033 mm. So that, our study did not encompass participants whose mean FD value was greater than 0.3mm. Consequently, the head motion of the participants included in our study remained within acceptable levels (27). The kurtosis and skewness of age (− 0.21, 0.06) and Raven scores (− 0.32, − 0.11) ranged from −1 to +1, indicating both age and intelligence data conformed to a normal distribution (28).

#### 2.5.1 Regional homogeneity-delay discounting correlation analysis

Regional Homogeneity (ReHo), a voxel-based measure of brain activity, operates on the assumption that the temporal pattern of a given voxel is akin to its neighboring voxels (29). This methodology aligns with the hypothesis that intrinsic brain activity is reflected by clusters of voxels rather than isolated voxels (30). In this study, we utilized the Kendall’s coefficient of concordance (KCC) as a metric to gauge ReHo (31). After controlling for age, sex, intelligence, and head motion parameters, we computed the correlation between the natural logarithm of *k* (Ln(*k*)) and ReHo, which measures the regional homogeneity of the whole-brain Blood-Oxygen-Level-Dependent (BOLD) signal. Multiple comparison correction in our study was conducted using the Gaussian Random Field theory (GRF) as implemented in the DPABI software (32). GRF is an established method in the domain of neuroimaging data analysis, particularly adept for handling 3D data. This approach is notable for its consideration of the spatial structure of neuroimaging data, which enhances the accuracy of multiple comparison corrections. For our analysis, we set the significance thresholds for both voxel level and cluster level evaluations at *p* < 0.05.

#### 2.5.2 Resting-state functional connectivity-delay discounting correlation analysis

Building upon the ReHo-delay discounting correlation analysis, we sought to further investigate the neural network associated with delay discounting during resting-state by employing resting-state functional connectivity (RSFC). Initially, the clusters identified in the ReHo-delay discounting correlation analysis were used as seeds. To ensure an adequate capture of the BOLD signal changes within the clusters, we created a sphere (radius of 5 mm) centered on the voxel demonstrating the most robust ReHo-delay discounting correlation within the cluster (33). The mean time series of the overlap between this cluster and the sphere were then extracted. Subsequently, we computed the functional connectivity by correlating the mean time series obtained from the seed with the time series of each voxel across the whole brain, while controlling for head motion, age, gender, and intelligence. Ultimately, for each seed, we identified the neural network involved in delay discounting by assessing the correlation between its functional connectivity and delay discounting for each voxel in the whole brain. In our analysis, the *r*-maps were converted into *T*-score maps. For the purpose of multiple comparison correction, we utilized the GRF as implemented in the DPABI software, following the guidelines of Yan et al. (32). We established the significance threshold for voxel level analysis at *p* < 0.05. Recognizing the importance of minimizing type I errors (false positives), a more stringent threshold of *p* < 0.01 was adopted at the cluster level. This approach aimed to achieve a balance between sensitivity and specificity in our statistical analysis, within the constraints of the current methodology and data.

## 3 Results

The delay discounting of the participants was denoted by the Ln(*k*) value, with higher Ln(*k*) values signaling elevated impulsivity levels, indicative of a preference for immediate and smaller rewards. The average Ln(*k*) for the participants was − 5.2, accompanied by a standard deviation of 1.53. The Ln(*k*) distribution followed a normal distribution pattern with a kurtosis of − 0.09 and skewness of − 0.07, both within the − 1 to 1 range. This attests to the reliability of using Ln(*k*) as a measure of delay discounting to probe the neural mechanisms of individuals in a resting state (28).

To investigate all brain regions implicated in delay discounting at a resting state, we computed the correlation between regional homogeneity (ReHo) and delay discounting in each voxel of the entire brain across all subjects. Post adjustment for head motion, age, and sex, we discovered a significant negative correlation of Ln(*k*) with two regions: the left insula (cluster size = 569; MNI coordinate: − 28, 0, − 16; GRF corrected, two tailed, voxel level *p* < 0.05, cluster level *p* < 0.05) and the right hippocampus (cluster size = 404; MNI coordinate: 16, − 14, − 16; GRF corrected, two tailed, voxel level *p* < 0.05, cluster level *p* < 0.05). This outcome implies that the insula and hippocampus contribute to individual differences in delay discounting at a resting state. More specifically, stronger ReHo of the insula and hippocampus translates to lower delay discounting, and an increased propensity to consider future choices of delayed rewards. In this analysis, no other significant results were identified.

**Figure 1:**
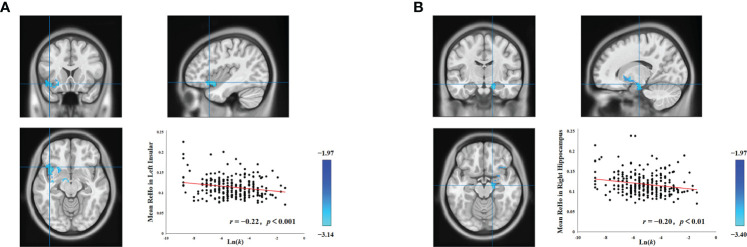
Associations between delay discounting and neural activity in the left insula and right hippocampus. **(A)** A cluster in the left insula exhibited a significant negative correlation between ReHo values and the Ln(*k*) values. **(B)** Similarly, a cluster in the right hippocampus also displayed a significant negative correlation between ReHo values and the Ln(*k*) values. For illustrative purposes, the scatter plot shows the correlations, adjusted for covariates including intelligence, age, gender, and head motion. Each data point represents an individual participant.

Individual intelligence is associated with the functioning of several brain regions, and both the hippocampus (34–37) and the insula (36, 38) have demonstrated associations with intelligence. Consequently, to control for the confounding influence of individual differences in intelligence on the associations between delay discounting and spontaneous brain activity in both the left insula and the right hippocampus, we performed supplementary analyses using intelligence as a covariate. Upon controlling for intelligence, head motion, age, and gender, we still observed a significant association between the left insula and delay discounting (cluster size = 570; MNI coordinate: − 40, 18, − 12; GRF corrected, two tailed, voxel level *p* < 0.05, cluster level *p* < 0.05; **Figure 1A**), and the right hippocampus and delay discounting (cluster size = 405; MNI coordinate: 16, − 14, − 16; GRF corrected, two tailed, voxel level *p* < 0.05, cluster level *p* < 0.05; **Figure 1B**). Such findings suggest that the relationship between the left insula and delay discounting, and the right hippocampus and delay discounting at a resting state is stable and is not attributed to variations in intelligence. Notably, the relationships between the left insula and delay discounting, as well as the right hippocampus and delay discounting, were not found to be statistically significant when analyzed using a permutation test with threshold-free cluster enhancement (PT TFCE), involving 1,000 permutations and adhering to a family-wise error rate (FWER) of less than 0.05.

**Figure 2:**
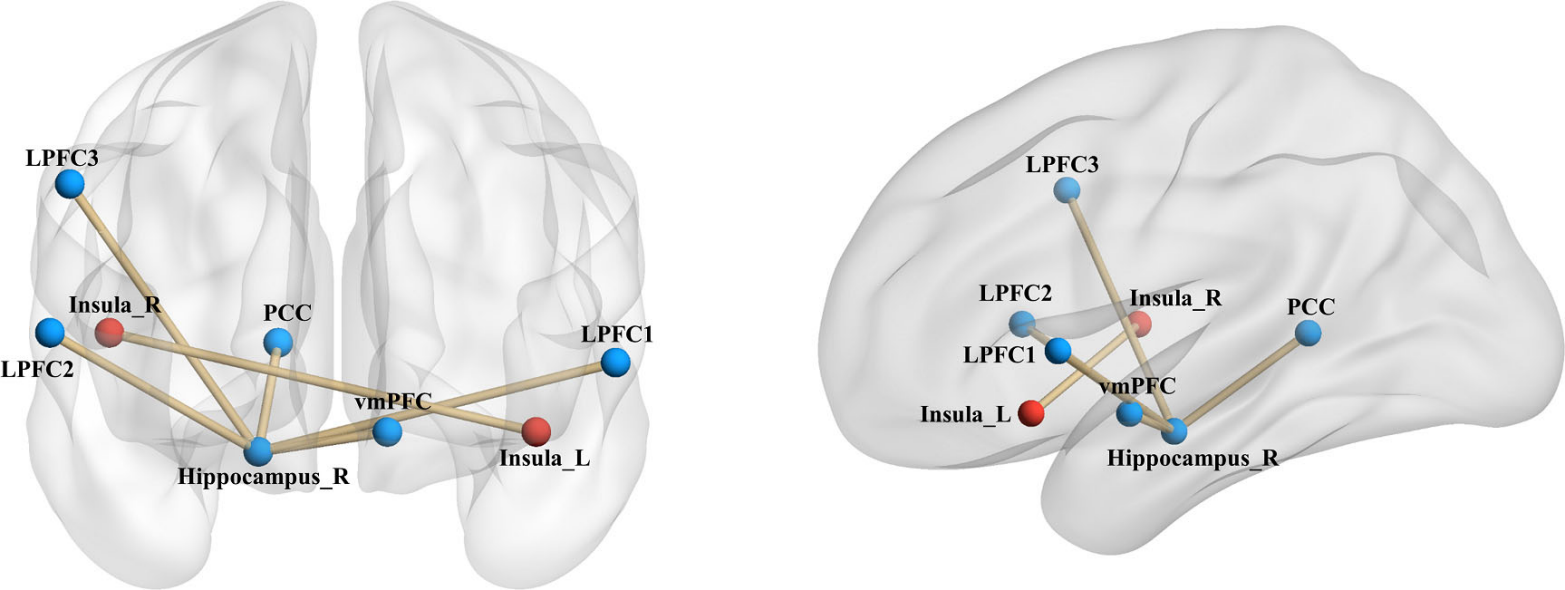
Seed-Based RSFC analysis highlighting associations with delay discounting. Utilizing the left insula and the right hippocampus as seed regions, distinct connectivity patterns were observed. The RSFC between the left insula and the right insula, denoted by red markers in the figure, was significantly positively correlated with the Ln(*k*) values. With respect to the right hippocampus as a seed region, its RSFC with both the vmPFC and the PCC demonstrated a significant negative correlation with the Ln(*k*) values. Conversely, connectivity with three clusters in the LPFC showed a significant positive correlation with the Ln(*k*), as indicated by blue markers in the figure. Nodes and edges representing these connectivity patterns are overlaid on inflated cortical surface maps, generated using BrainNet Viewer (39).

Upon establishing the roles of the left insula and the right hippocampus in delay discounting during resting states, we used these ROIs separately as seed regions to investigate the neural network associated with delay discounting via whole-brain resting-state functional connectivity (RSFC). Specifically, we assessed the correlation between the Ln(*k*) values and RSFC for each voxel relative to the left insula and right hippocampus across all participants. These analyses were conducted after controlling for variables such as intelligence, age, gender, and head motion. We found that the RSFC between the left insula and the right insula was significantly positively correlated with participants’ Ln(*k*) values (*r* = 0.20, *p* < 0.01; **Table 1**, **Figure 2**; the right insula cluster size: 560; MNI coordinate: 46, − 6, 8; GRF corrected, two tailed, voxel level *p* < 0.05, cluster level *p* < 0.01). Specifically, increased RSFC between the left and right insula was associated with higher Ln(*k*) values, indicating a greater tendency for participants to opt for smaller, immediate rewards (SIRs) due to a steeper discounting of future values. Conversely, when the right hippocampus served as the seed ROI, we identified significant positive or negative correlations between the RSFC of the right hippocampus and five discrete clusters in the vmPFC, PCC, and LPFC with participants’ Ln(*k*) values, as illustrated in **Table 1**; **Figure 2**. These results underscore the modulatory effect of resting-state functional connectivity among these neural regions on individuals’ delay discounting.

**Table 1:** Correlations between RSFC and delay discounting.

RSFC	Cluster size (voxels)	Peak T score	MNI coordinate			Statistic	Correlation coefficient
			x	y	z		
Insula_L-Insula_R	560	3.24	46	− 6	8	p<0.01, corrected	0.20
Hippocampus-vmPFC	1038	− 4.19	− 10	− 4	− 12	p<0.001, corrected	− 0.25
Hippocampus-PCC	1663	− 3.63	12	− 44	6	p<0.001, corrected	− 0.23
Hippocampus-LPFC1	657	3.79	− 56	12	2	p<0.001, corrected	0.23
Hippocampus-LPFC2	921	3.71	58	20	8	p<0.001, corrected	0.25
Hippocampus-LPFC3	689	4.64	54	10	38	p<0.001, corrected	0.28

## 4 Discussion

This study employed rs-fMRI to probe the brain networks involved in individual differences in delay discounting, using a substantial sample of healthy university students. Initially, we observed the significant negative correlations between regional homogeneity (ReHo) and delay discounting, particularly within the left insula and the right hippocampus. Stronger regional homogeneity of spontaneous activity in these brain regions was associated with lower delay discounting, implying a higher likelihood of choosing delayed rewards. Further exploration of the neural networks involved in delay discounting at rest was conducted using the left insula and the right hippocampus as seed regions. Interestingly, all the brain regions implicated in individual differences in delay discounting that we identified in the resting-state functional connectivity (RSFC) analysis belong to the three principal networks: the valuation, cognitive control, and prospection networks. This study is the first to elucidate, from the perspective of spontaneous neural activity, how these three neural networks interact with each other to influence individual variability in delay discounting.

**Figure 3:**
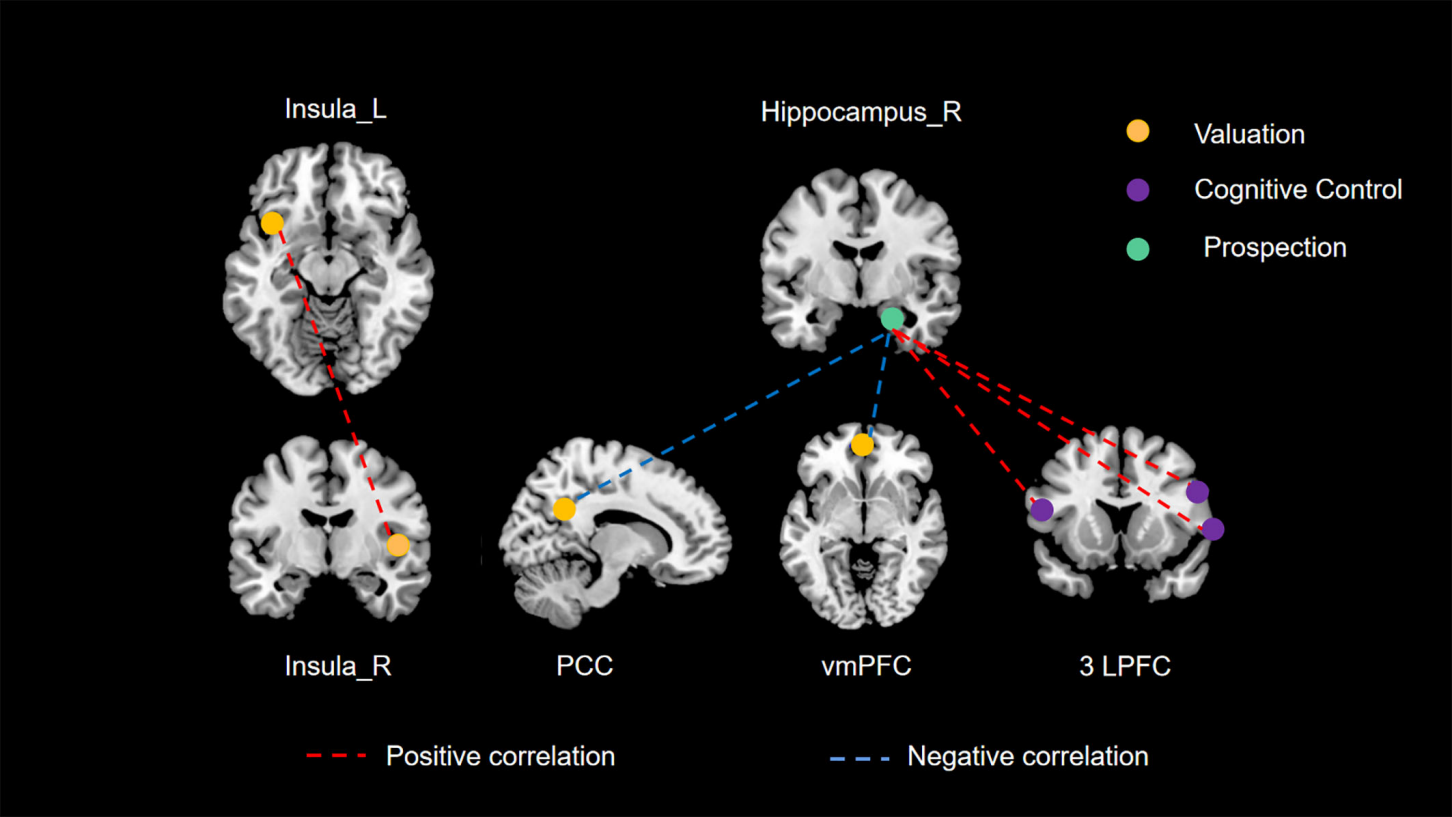
The three principal networks underlying delay discounting. This illustration delineates the three principal networks implicated in delay discounting: the valuation network (represented in yellow), the cognitive control network (represented in purple), and the prospection network (represented in green). The various colored dashed lines signify distinct relationships between resting-state functional connectivity (RSFC) and delay discounting. Specifically, red dashed lines denote significant positive correlations between functional connectivity and delay discounting across different brain regions, whereas blue dashed lines illustrate significant negative correlations.

Drawing on empirical evidence from cognitive neuroscience methodologies such as fMRI, previous research has posited that three principal neural networks (valuation, cognitive control, and prospection) are predominantly engaged in modulating delay discounting behavior (40–42). Moreover, Mehta et al. (43) conducted a connectome-wide association study (CWAS) to explore the interplay between RSFC and delay discounting in a demographic spanning 9 to 23 years. Their study pinpointed the posterior cingulate cortex (PCC), ventromedial prefrontal cortex (vmPFC), and lateral prefrontal cortex (LPFC) as crucial elements of these neural networks, substantially involved in delay discounting. Such findings provide additional validation for our research. Nonetheless, there remains a notable gap in empirical literature concerning the validation of this tripartite neural network framework in the context of spontaneous neural activity. Our current study aims to bridge this gap. Employing comprehensive whole-brain analyses at both regional and network scales, it is noteworthy that all identified brain regions associated with delay discounting fell exclusively within these three principal networks, as illustrated in **Figure 3**. Particularly, our study’s unique contribution is in delineating the interconnections among these principal networks through an RSFC analysis.

Firstly, in our study, we found a significant positive correlation between the RSFC of the left and right insula and delay discounting behaviors. The insula is a crucial component of the valuation network, which involves several brain regions responsible for evaluating and appraising the intrinsic value of rewards (9, 44, 45). Increased activity within this network has been linked to impulsive choices and behaviors (7). Numerous studies have emphasized the insula’s significant role in the valuation process during decision-making (46, 47). In this context, enhanced RSFC between the left and right insular regions may suggest an increased integration and coordination within the valuation network. This augmented connectivity could potentially lead to a more efficient and rapid assessment of rewards, thus facilitating quicker response to reward-related stimuli. Consequently, this heightened state of responsiveness in the valuation network, as a result of increased insula connectivity, could predispose individuals to more impulsive decision-making. This is because the amplified insula communication may accelerate the valuation process, making immediate rewards appear more salient and desirable, and thus skewing choices towards impulsivity. This effect might overshadow the consideration of long-term outcomes, leading to a preference for immediate gratification over delayed rewards.

Secondly, the hippocampus, a central structure in the prospection network, interacts with the valuation and cognitive control networks. The ventromedial prefrontal cortex (vmPFC) and posterior cingulate cortex (PCC) are core regions within the valuation network, playing a role in individual value representation (48–52). The hippocampus-dominated prospection network is thought to be closely intertwined with the valuation network, as certain areas within the valuation network also belong to the prospection network (7). The prospection network is concerned with future planning and the projection of oneself into future scenarios. Consequently, the right hippocampus-vmPFC RSFC and right hippocampus-PCC RSFC may reflect the cooperative function of the prospection and valuation networks. Our study reveals that a stronger connectivity between the prospection network and the valuation network is associated with a greater propensity for individuals to opt for delayed rewards. This may be attributed to the prospection network’s emphasis on the future consequences of current choices (53, 54). Enhanced connectivity with the valuation network appears to inhibit the latter’s facilitative effect on impulsive choices, thereby aiding individuals in making decisions that favor long-term benefits over immediate gratification.

Furthermore, the lateral prefrontal cortex (LPFC), a crucial component of the cognitive control network, plays a pivotal role in managing decision-making complexities, particularly when choices bear comparable values (7). It primarily functions as a conflict monitor, aiding in the resolution of decision-making conflicts (55, 56). In our study, we observed a positive correlation between the RSFC of the right hippocampus and all three LPFC clusters in relation to delay discounting. This suggests that stronger RSFC between the right hippocampus and these LPFC clusters is associated with increased impulsivity. To elucidate further, the hippocampus is integral in encoding and retrieving memories (57, 58), and its interaction with the LPFC is critical for integrating past experiences into current decision-making processes. When the RSFC between these regions is enhanced, it could imply an overemphasis on immediate experiences or rewards at the expense of long-term considerations. This heightened connectivity may lead to a dominance of immediate rewards in decision-making, overshadowing the rational evaluation of delayed outcomes typically modulated by the LPFC. Consequently, this imbalance, where the cognitive control exerted by the LPFC is compromised by the amplified influence of the hippocampus, may result in a bias towards impulsive choices. This scenario highlights a potential mechanism whereby increased hippocampal-LPFC connectivity disrupts the equilibrium between immediate and future reward evaluation, steering decisions towards impulsivity.

Methodologically, our study supplements existing research on the neural networks involved in delay discounting during rest in three significant ways. Firstly, capitalizing on our substantial participant sample, we executed a whole-brain analysis at both the regional and neural network levels. This diverges from the common practice of merely selecting specific regions of interest for exploration (10, 59–63). A whole-brain-based analysis allows for a more comprehensive identification of the neural networks implicated in delay discounting. Secondly, in the computation of functional connectivity, we determined the mean time series within the region where regional homogeneity (ReHo) demonstrated the strongest correlation with delay discounting. We centered a 5-mm radius sphere in this region (33, 64–67). This method enhances the representativeness of our results and provides a valuable contribution to the study of neural networks involved in delay discounting. Thirdly, we considered the established connection between individual delay discounting and intelligence, where individuals with lower intelligence exhibit higher delay discounting (68, 69). Consequently, we controlled for the influence of intelligence when computing correlations, thus lending greater credibility to our findings.

However, this study also has certain limitations. Firstly, a significant limitation of our study pertains to the reliance on the threshold level selected for multiple comparisons correction. While our results demonstrated significance under more liberal thresholds, they did not sustain this significance when subjected to more stringent multiple comparisons correction. Specifically, at both the regional and network levels, the outcomes did not meet the rigorous voxel-level threshold of less than 0.01 (GRF correction). Additionally, our results were also unable to pass the permutation test with a *p*-value threshold of less than 0.05. This limitation accentuates a core challenge in neuroimaging research: balancing the identification of true effects (sensitivity) against the prevention of false positives (specificity). The employment of stringent thresholds, aimed at reducing false positive rates, concurrently elevates the risk of missing true small effect sizes, which were the primary focus of our investigation. Moreover, our adoption of a whole-brain correction approach, while methodologically rigorous, encountered difficulties in detecting small effect sizes due to the extensive correction base. This methodological choice necessitates a cautious interpretation of our findings, particularly concerning their replicability and applicability to other studies and diverse populations. It is crucial to consider these factors when evaluating the implications and generalizability of our research findings within the broader scientific context. Secondly, in our examination of the role of spontaneous neural activity in shaping individual variations in delay discounting at both regional and network levels, one notable limitation is the exclusion of socioeconomic status (SES) as a variable. Prior studies have highlighted the relevance of both subjective and objective SES in influencing delay discounting behaviors. For instance, research has shown that individuals with a higher perceived social status (subjective SES) tend to exhibit lower levels of delay discounting (70, 71). Similarly, objective measures of SES, such as parental education levels, have been linked to variations in delay discounting, as demonstrated in studies like Mehta et al. (43). Our study’s focus on neural mechanisms did not encompass the potential modulatory effects of SES, which could be a significant factor in delay discounting. Recognizing this, we suggest that future investigations in this field would benefit from integrating SES into their analytical frameworks to provide a more comprehensive understanding of the neural underpinnings of delay discounting.

In summary, by integrating results at the regional and neural network levels, this study contributes preliminary evidence illuminating how the valuation network, the cognitive control network, and the prospection network interact during resting state to influence individual delay discounting. Future research should delve deeper into this area. Firstly, although our whole-brain analysis only implicated certain brain regions within the three networks, this does not preclude the potential involvement of other brain regions within these networks. Their effects might approach statistical significance but fall short of our current threshold. Therefore, future investigations might select regions of interest within these three networks for in-depth examination. Secondly, our participant cohort comprised healthy university students. Future research could consider extending the focus to groups characterized by high impulsivity levels (i.e., high delay discounting), such as individuals with addiction. Insights at the neural level from such studies could be valuable for devising effective intervention and treatment strategies. Thirdly, delay discounting is traditionally utilized as a method for measuring impulsivity (72, 73). Importantly, prior research has identified a relationship between impulsivity, as measured by the Barratt Impulsiveness Scale, and in-scanner motion. Therefore, future research endeavors could be directed towards exploring delay discounting as a behavioral indicator of impulsivity. This would particularly include probing whether increased impulsivity, as manifested in delay discounting tasks, is associated with elevated in-scanner motion.

## Data availability statement

The raw data supporting the conclusions of this article will be made available by the authors, without undue reservation.

## Ethics statement

The studies involving humans were approved by Institutional Review Board of Beijing Normal University in Beijing, China. The studies were conducted in accordance with the local legislation and institutional requirements. The participants provided their written informed consent to participate in this study.

## Author contributions

SJ: Writing – original draft, Data curation. FY: Data curation, Writing – original draft. XL: Conceptualization, Writing – review & editing.
